# 

**Published:** 1871-07

**Authors:** 


### ARTICLE III.

### *American Dental Association.*

By M. S. Dean, Secretary.

The 11th annual meeting of the American Dental Association, will be held at White Sulphur Springs Va., commencing at 10 o'clock in the morning, on Tuesday the 1st day of

August next. The following facts are gathered from a pamphlet by Prof. Moorman, a resident of that place.

"White Sulphur Springs is situated in Greenbrier County, on the western slope of the great Apalachian chain of mountains." Latitude 37½° North, Longitude 3½° west of Washington. "The Thermometer ranges in Summer, between 55° and 65°, and rarely attains a greater height than 80° at any time in the day." The situation of the springs is elevated, and beautifully picturesque, surrounded on all sides by mountains. The Springs are at the present terminus of the Chesapeake and Ohio railroad. The traveler by rail to these, ( from any quarter of the country,) must pass through the town of Staunton.

Persons from the East, North or West, make Baltimore or Washington, points in their route to the Springs. At Washington they take the Orange and Alexandria road to Gordonsville ; then the Chesapeake and Ohio, to White Sulphur by Staunton. Those from the South have a continuous line of railroad, either by Richmond Va., or Knoxville Tenn. Persons going by the Knoxville route, will take the Virginia and Tennessee railroad to Lynchburg ; then the Orange and Alexandria to Charlottesville ; then the Chesapeake and Ohio to the Springs. Those taking the Knoxville route, will find a daily line of stages at the "Montgom ery White," that run to White Sulphur, a distance of sixty-five miles. Accommodations at White Sulphur, seem to be ample. "The hotel building" covering more than an acre of ground. The dining-room conveniently seating twelve hundred persons."

The committee to secure reduced fare to and from the place of meeting, are actively engaged, and will be heard from soon.

---

### ARTICLE IV.

### *American Dental Convention.*

The undersigned Committee of Arrangements would inform the *dental profession* that they have made arrangements for holding the seventeenth annual meeting of the American Dental Convention at Saratoga Springs, commencing on Wednesday the 9th day of August, at 11 A. M.

Should the attendance be as large as anticipated, a liberal deduction will be made to members of the convention by the proprietors of the two principal hotels, with the assurance that no effort will be spared to make their guests as comfortable as possible.

Arrangements will be made for the display and exhibition of dental materials and appliances of all kinds. Also for clinical operations, during the session.

Dentists and others desirous of exhibiting instruments, materials or improvements in any department of dentistry, are particularly invited to present them as early in the session as possible, and to notify the committee of their intention to do so.

As August is a very busy month with hotel keepers, it will be advisable for those intending to be present to notify the committee of their wishes &c., at 25 West 23rd St., New York.

Efforts are making to secure a reduction of fare by rail and steam boat.

J. G. Ambler, J. S. Lattimer, W. B. Hurd, J. S. Smith,
*Committee of Arrangements.*

---

